# Hi-C guided assemblies reveal conserved regulatory topologies on X and autosomes despite extensive genome shuffling

**DOI:** 10.1101/gad.328971.119

**Published:** 2019-11-01

**Authors:** Gina Renschler, Gautier Richard, Claudia Isabelle Keller Valsecchi, Sarah Toscano, Laura Arrigoni, Fidel Ramírez, Asifa Akhtar

**Affiliations:** 1 Max Planck Institute of Immunobiology and Epigenetics, 79108 Freiburg im Breisgau, Germany;; 2 Faculty of Biology, University of Freiburg, 79104 Freiburg, Germany;; 3 IGEPP, INRA, Agrocampus Ouest, Université Rennes, 35600 Le Rheu, France

**Keywords:** dosage compensation, HiC, X chromosome, chromosome topology

## Abstract

Genome rearrangements that occur during evolution impose major challenges on regulatory mechanisms that rely on three-dimensional genome architecture. Here, we developed a scaffolding algorithm and generated chromosome-length assemblies from Hi-C data for studying genome topology in three distantly related *Drosophila* species. We observe extensive genome shuffling between these species with one synteny breakpoint after approximately every six genes. A/B compartments, a set of large gene-dense topologically associating domains (TADs), and spatial contacts between high-affinity sites (HAS) located on the X chromosome are maintained over 40 million years, indicating architectural conservation at various hierarchies. Evolutionary conserved genes cluster in the vicinity of HAS, while HAS locations appear evolutionarily flexible, thus uncoupling functional requirement of dosage compensation from individual positions on the linear X chromosome. Therefore, 3D architecture is preserved even in scenarios of thousands of rearrangements highlighting its relevance for essential processes such as dosage compensation of the X chromosome.

## Abstract (precis)

In this study, Renschler et al. set out to analyze the impact of genomic rearrangements on genome topology using the Drosophila genus and X chromosome dosage compensation as a model. The authors developed a scaffolding algorithm and generated chromosome-length assemblies from Hi-C data for studying genome topology in three distantly related Drosophila species. Their data provides unique insights into genome topology evolution. RA

Chromosome conformation capture techniques such as Hi-C provide genome-wide contact maps between loci within chromosomes (Lieberman-Aiden et al. 2009). Those techniques revealed several regulatory layers of genome organization, including regions that show preferential contacts within them referred to as topologically associating domains (TADs) (Dixon et al. 2012; Nora et al. 2012). TADs and their boundaries tend to correlate with genomic rearrangements during evolution as interspecies comparisons in mammals revealed few selected examples in which contiguous orthologous genes inserted at different genomic positions in a different species maintain TAD integrity (Vietri Rudan et al. 2015). TADs have also been described in non-mammalian species (e.g., *Drosophila*) (Sexton et al. 2012) whose genome is more than 10-times smaller, more gene-dense, and exposed to faster rates of molecular evolution compared to mammals (Thomas et al. 2010). It remains unclear whether genome architecture is maintained in highly rearranged, yet related, genomes within a given genus such as *Drosophila*.

Except for *D. melanogaster*, current *Drosophila* genome assemblies (Drosophila 12 Genomes Consortium et al. 2007; Wiegmann and Richards 2018) are typically composed of thousands of scaffolds, which hinder comparisons related to genome organization. Analyzing genomic rearrangements and their impact on 3D genome architecture requires chromosome-length genome assemblies. Hi-C-derived information can aid for such questions, because contacts between pairs of loci in the whole genome provide linking information to order and orient genome scaffolds into entire chromosomes. Prime examples of such Hi-C-assisted genome assemblies are the mosquito *Aedes aegypti*, the domestic goat *Capra hircus*, or the barley *Hordeum vulgare L*. (Burton et al. 2013; Kaplan and Dekker 2013; Korbel and Lee 2013; Marie-Nelly et al. 2014; Bickhart et al. 2017; Dudchenko et al. 2017; Mascher et al. 2017). Apart from being cost-effective, such assemblies at the same time provide additional information about genome conformation.

The observation of genomic rearrangements throughout evolution can raise the question of how they impact mechanisms of transcriptional regulation at genome-wide or chromosome-wide scales. One example of such a chromosome-wide process is dosage compensation, which balances the transcriptional output from sex chromosomes between males and females. In *D. melanogaster*, the Male-specific lethal (MSL) complex mediates approximately twofold up-regulation of X-linked genes in males (Kuroda et al. 2016; Samata and Akhtar 2018), and this appears to be conserved in other drosophilids (Russo et al. 1995; Robe et al. 2010; Alekseyenko et al. 2013; Quinn et al. 2016). In *D. melanogaster*, the X chromosome adopts a dedicated 3D architecture, where X-linked recruitment sites for the MSL complex, termed high-affinity sites (HAS), are enriched in Hi-C contacts and appear to cluster in space (Ramírez et al. 2015; Schauer et al. 2017).

To study these questions, we generated Hi-C data of *D. melanogaster*, *D. virilis*, and *D. busckii* embryos and assembled chromosome-length genomes of the latter two species. We choose to study *D. virilis* and *D. busckii* based on the phylogenetic position of the two species in the *Drosophila* genus, as they cover ∼40 million years of evolution and multiple subgenera (Russo et al. 2013). Because of their evolutionary distance, but similar functional and developmental constraints, these species provide an exciting model system to study highly rearranged, yet related, genomes within a given genus. Indeed, experimental mapping of the HAS positions on the X chromosome by roX ChIRP-seq revealed that the individual HAS positions undergo rapid evolutionary turnover (Quinn et al. 2016). However, it remained unclear how this would impact their interactions and the 3D conformation of the X chromosome, particularly in light of the extensive genomic rearrangements occurring in these species. We developed HiCAssembler, a Hi-C scaffolding tool allowing the assembly of genomes using Hi-C data combined with scaffolds obtained from short- and long-read sequencing that is compatible with our previously published package for Hi-C data processing, HiCExplorer (Ramírez et al. 2018). Using these data and tools, we find extensive rearrangements within chromosomes, whereas higher-order genome topology (A/B compartments) and a subset of TADs appear to be maintained as conserved units. Underscoring the functional relevance of maintaining genome topology, we find that spatial contacts implicated in X chromosome dosage compensation are preserved over millions of years of evolution and suggest that they are not a mere consequence of closeness to TAD boundaries or the expression level of their associated genes. Our study in these highly rearranged genomes highlights the importance of maintaining genome topology during evolution, which may shape even chromosome-wide regulatory mechanisms such as on the X chromosome.

## Results

### Chromosome-length assemblies of the *D. busckii* and *D. virilis* genomes

**Figure 1.**
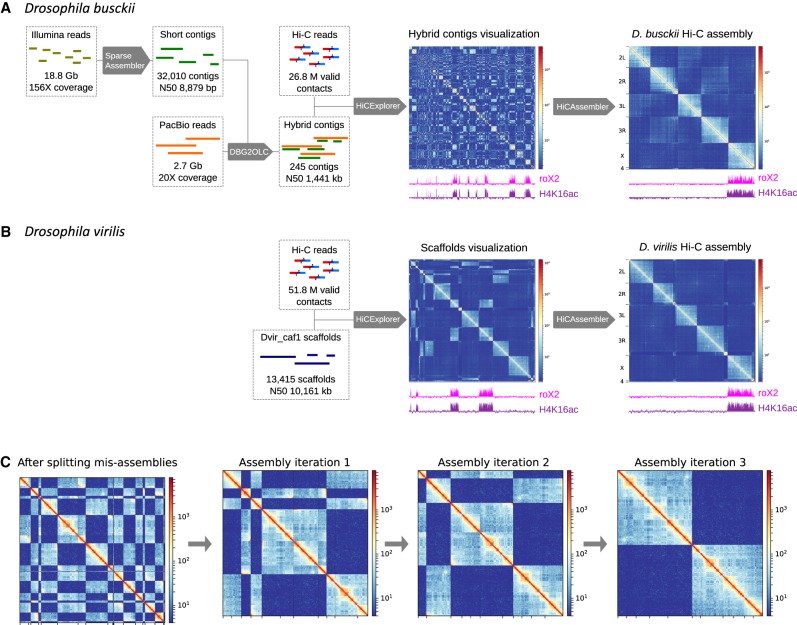
Hi-C guided chromosome-length assemblies of *D. busckii* and *D. virilis* genomes. (*A*) De novo assembly of *D. busckii* genome. A hybrid approach integrating long PacBio reads and short contigs assembled from Illumina reads was used to obtain 245 de novo contigs of the *D. busckii* genome. Assembly of 156× Illumina reads using SparseAssembler (Ye et al. 2012) resulted in 32,010 short contigs. 20× PacBio data was integrated using DBG2OLC (Ye et al. 2016), which increased the N50 more than 100-fold. These 245 hybrid contigs were scaffolded into chromosome-length with Hi-C data using HiCAssembler. Integrity of the X chromosome (identified by whole-genome alignment to *D. melanogaster*) was validated using ChIRP-seq data of the dosage compensation complex member roX2 (Quinn et al. 2016) and ChIP-seq data of H4K16ac from male *D. busckii* larvae. (*B*) *D. virilis* Hi-C assembly. The existing reference scaffolds of *D. virilis* (Dvir_caf1 scaffolds) were assembled into full chromosomes using HiCAssembler. The enrichment of roX2 and H4K16ac (male) on one chromosome depicts full integrity of the assembled X chromosome. (*C*) Overview of HiCAssembler strategy (see Materials and Methods and Supplemental Fig. S3 for a complete description of the algorithm). The figure displays the iterative progression of the Hi-C assembly strategy as in Dudchenko et al. (2017) for a small example Hi-C matrix. First, the original scaffolds are split if they contain misassemblies and small scaffolds are removed. In each iteration of the Hi-C assembly algorithm scaffolds are joined and oriented to form larger and larger Hi-C scaffolds until chromosome-length assemblies are obtained as shown in the last panel where two separated blocks remain. Afterward, the small scaffolds that were initially removed are inserted into the Hi-C scaffolds.

To study the impact of chromosome rearrangements on genome topology, we generated in situ Hi-C data from *D. melanogaster*, *D. busckii*, and *D. virilis* mixed-sex embryos at stage 15–16. We then used this data to generate chromosome-length genome assemblies of *D. virilis* and *D. busckii* (Fig. 1). For *D. virilis*, we used sequence information from the *Drosophila* 12 Genomes Consortium (*Drosophila* 12 Genomes Consortium et al. 2007), which contains 13,415 scaffolds (Dvir_caf1, N50 = 10.2 Mb). For *D. busckii*, we integrated previously published short Illumina reads (Vicoso and Bachtrog 2015; Zhou and Bachtrog 2015) that we assembled into 32,010 short-read contigs using SparseAssembler (Ye et al. 2012). We then generated 2.7 Gb (∼20× genome coverage) PacBio reads of *D. busckii* gDNA (Supplemental Fig. S1). The Illumina reads were combined with the error-corrected PacBio read data using DBG2OLC (Ye et al. 2016) to obtain a total of 245 longer contigs with an N50 of 1.4 Mb (Table 1).

**Table 1.**
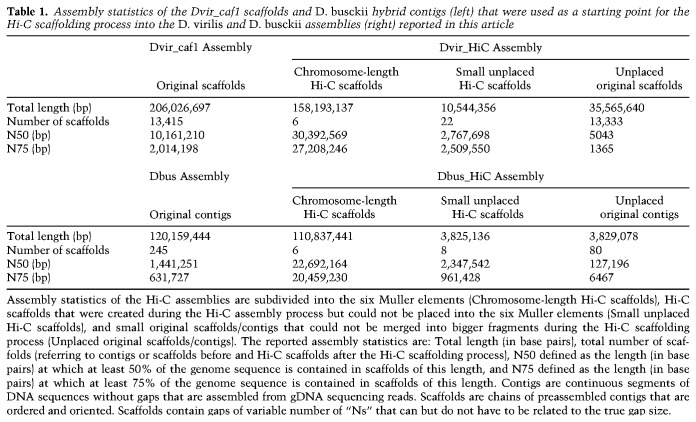
Assembly statistics of the Dvir_caf1 scaffolds and *D. busckii* hybrid contigs (left) that were used as a starting point for the Hi-C scaffolding process into the *D. virilis* and *D. busckii* assemblies (right) reported in this article

We then used the Hi-C data to produce chromosome-length assemblies of the *D. busckii* and *D. virilis* genomes. For this, we developed the algorithm “HiCAssembler,” which uses strategies derived from LACHESIS (Korbel and Lee 2013) and 3D-DNA (Dudchenko et al. 2017) and is freely available at https://github.com/maxplanck-ie/HiCAssembler.

HiCAssembler uses the linking information from Hi-C contacts to order preassembled contigs/scaffolds depending on their contact frequency. This is possible because the Hi-C contact frequency follows a power-law decay by distance (Lieberman-Aiden et al. 2009) (i.e., the linear proximity of contigs/scaffolds can be inferred from Hi-C data and used to assemble them into entire chromosomes). In brief, a Hi-C matrix is created by aligning the Hi-C reads to the pre-assembled contigs/scaffolds. Then, small fragments (default parameter of 150 kb) are put aside and the original contigs/scaffolds are split, if they contain misassemblies. Such misassemblies can be detected as regions in the Hi-C matrix that do not follow the power-law decay with respect to genomic distance and are easy to spot as discontinuous regions in the Hi-C signal (Supplemental Fig. S3E). HiCAssembler provides both a computational and a manual method to detect misassemblies. In each iteration of the Hi-C assembly algorithm, scaffolds are joined and oriented to form larger Hi-C scaffolds until chromosome-length assemblies are obtained (Fig. 1C). Afterward, initially removed small fragments are inserted into the Hi-C scaffolds (see Supplemental Fig. S3 and the Materials and Methods section for a detailed description of HiCAssembler, and Supplemental Table S1 for a comparison with other Hi-C scaffolding tools).

The resulting chromosome-length assemblies of 118.5 Gb (*D. busckii*) or 204.3 Gb (*D. virilis*) consist of chrX, chr2L, chr2R, chr3L, chr3R, and chr4 corresponding to the Muller elements A–F (Table 1; Fig. 1; Supplemental Fig. S2A; Muller 1940). Yet, both assemblies contain additional “unplaced Hi-C scaffolds,” which correspond to original contigs/scaffolds that were joined into bigger fragments as well as “unplaced original contigs/scaffolds” that could not be assembled into bigger fragments by Hi-C scaffolding (Table 1).

For validation, we generated H4K16ac ChIP-seq of separated male and female larvae and aligned ChIRP-seq of roX2 (Quinn et al. 2016) to our assemblies, as, based on their roles in X chromosome dosage compensation, they are expected to be enriched on the male X. We observe H4K16ac and roX2 enrichment only in one male chromosome-length scaffold, which is present in a single copy in males and two copies in females, and hence, corresponds to the X chromosome (Supplemental Fig. S2C,D). This underscores the major improvement in terms of continuity over previous genome assemblies, in which roX2 and H4K16ac are scattered across numerous scaffolds (Fig. 1A,B). Additionally, we confirmed the quality of our *D. busckii* and *D. virilis* assemblies with Benchmarking Universal Single-Copy Orthologs (BUSCOs) (Waterhouse et al. 2017), which are sets of genes with single-copy orthologs in >90% of selected species. BUSCOs can be used to quantitatively measure completeness of genome assemblies (Simão et al. 2015). The *Diptera* (odb9) data set contains 2799 BUSCOs, and we detected 95.7% and 98.1% complete BUSCOs in *D. busckii* and *D. virilis*, respectively (Supplemental Fig. S2E). Such high BUSCO scores emphasize the quality and completeness of our HiCAssembler-generated chromosome-length genome assemblies of *D. busckii* and *D. virilis*, allowing us to draw valid conclusions about genome topology and evolution in the *Drosophila* genus.

### Conserved TADs are shuffled along the genome during *Drosophila* evolution

**Figure 2.**
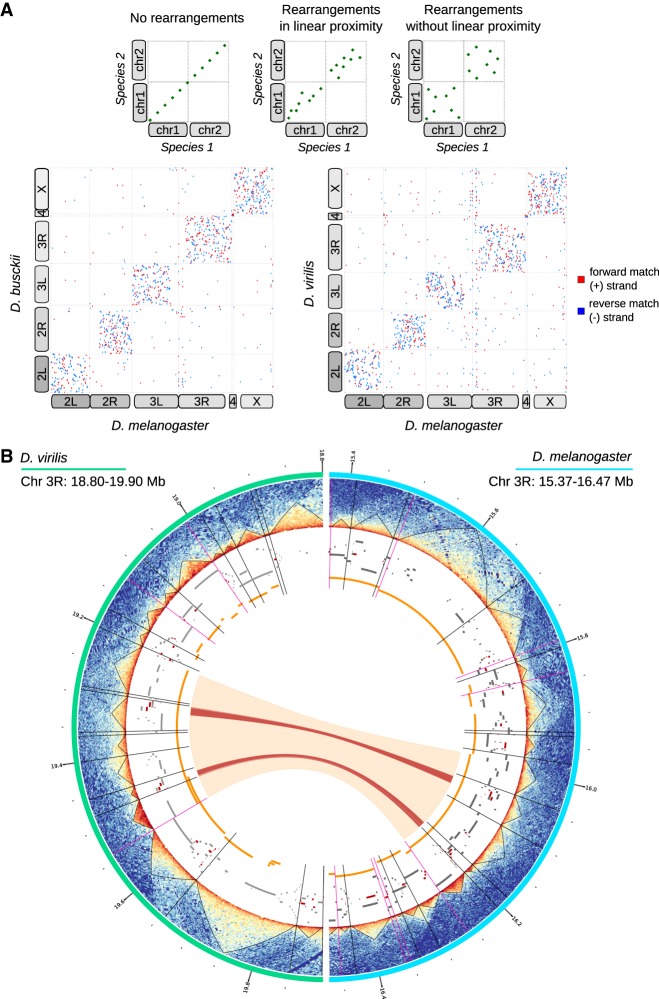
Extensive genome shuffling during *Drosophila* evolution. (*A*, *top*) Hypothetical whole-genome alignments. If no rearrangements have occurred between two species, whole-genome alignments result in matches that perfectly align at the diagonal (*left*). If there was a link between linear proximity and synteny breakpoints, matches would be expected to converge near the diagonal (*middle*). If shuffling happens without linear proximity, matches would occur randomly throughout the whole-chromosome arms (*right*). (*Bottom*) Dotplots showing actual whole-genome alignments between *D. melanogaster* and *D. busckii* or *D. virilis*, respectively. Alignments were performed using Mummer4 (Marçais et al. 2018). Forward matches (+ strand) are shown in red; reverse matches (− strand) are displayed in blue. Corresponding chromosome arms are indicated with boxes that are displayed connected if chromosome arms are fused in one species. Karyotypes are additionally depicted in Supplemental Figure S2A. (*B*) Association between TAD boundaries and synteny block breakpoints. From the exterior to the interior of the Circos plot. (Turquoise) The 18.80–19.90 Mb region of the chromosome 3L in *D. virilis*, (light blue) the 15.37–16.47 Mb of the chromosome 3L in *D. melanogaster*, (heatmaps) Hi-C contact heatmaps with TADs displayed as black triangles, (black radial lines) TAD boundaries, (magenta radial lines) TAD boundaries overlapping with synteny block start or end sites, (gray blocks) genes, (red blocks) BUSCOs, (orange blocks) synteny blocks, (orange arc) conserved synteny block between *D. virilis* and *D. melanogaster* in the displayed regions, (red arcs) conserved BUSCOs in the displayed regions.

Pairwise comparisons between these genome assemblies of *D. virilis* and *D. busckii* with *D. melanogaster* revealed extensive genomic rearrangements between the three species (Fig. 2A). We observed that conserved sequences mostly reside on the same chromosomal arms, whereas only few conserved sequences are found between two different arms. Rearrangements arise without any particular orientation preference. Moreover, shuffling occurs throughout the entire chromosomal arms in the absence of any apparent pattern concerning proximity or distance on the linear DNA sequence. Quantification of the density of synteny breakpoints within all chromosomes supports this observation (Supplemental Fig. S5A).

We next analyzed a potential connection between genomic rearrangements occurring during evolution and the 3D architecture of the *D. melanogaster*, *D. busckii*, and *D. virilis* genomes. For this we defined synteny blocks (SBs), which are chains of conserved collinear regions that are used to identify and compare homologous regions between different species. On average, we find 20 synteny breakpoints per megabase (3726 and 3252 breakpoints in the *D. melanogaster* vs. *D. virilis* comparison, respectively, and 3340 and 2776 breakpoints in the *D. melanogaster* vs. *D. busckii* comparison, respectively; see Materials and Methods), corresponding to about one breakpoint every six genes. We then compared SBs with two genome topology hierarchies, active/inactive compartments (A/B compartments) and TADs. After obtaining A/B compartments at ∼25-kb resolution from the Hi-C data in all three species (see Materials and Methods), we correlated the first eigenvector (PC1) of corresponding SBs. We find an *r* = 0.45 for *D. melanogaster* and *D. virilis* and *r* = 0.42 for *D. melanogaster* and *D. busckii.* Compared with this relatively high correlation between corresponding SBs, random SBs show no correlation with the actual SBs (*r* = −0.04 and *r* = −0.06) and SBs between two data sets from the same species show very high correlation (*r* = 0.93) (Supplemental Fig. S5B,C). Approximately 75% of SBs stay within the A or B compartment and 25% switch between compartments (Supplemental Fig. S5D). In general, about double the number of SBs lie within the A compartment than the B compartment. Therefore, higher-order genome topology (A/B compartments), especially the active compartment, appears to be maintained over 40 million years of genome reshuffling.

We next called TADs in our Hi-C data sets at restriction fragment resolution and validated our TAD calling using several metrics, including a comparison with data that has been sequenced at 10-fold higher sequencing depth (Eagen et al. 2017). We also compared our TAD positions (called using HiCExplorer) with the TAD positions reported by Eagen et al. (2017) (called using Arrowhead) (Durand et al. 2016), which showed great agreement as indicated by ChIP enrichment of the common insulator protein cofactor CP190 (Li et al. 2015). As histone modifications are known to correlate within TADs, we additionally used the H4K16ac ChIP-seq data obtained in all three species to further validate TAD positions (Supplemental Fig. S4). Using these different metrics, we found that our TADs are comparable to the ones reported by Eagen et al. (2017) and allow us, despite the lower sequencing depth, to draw valid conclusions about TAD evolution.

**Figure 3.**
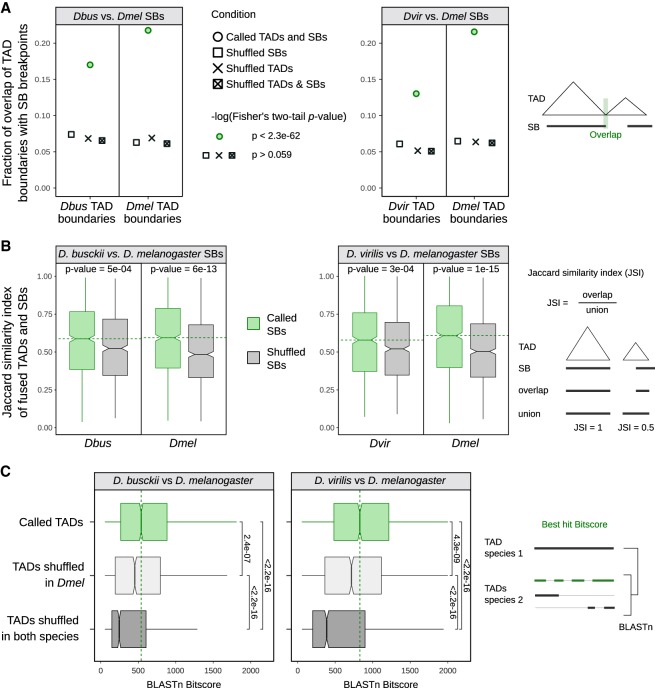
TAD boundaries correlate significantly with synteny block breakpoints. (*A*) Fraction of overlapping extended TAD boundaries with extended synteny block (SB) start and end sites in comparisons of *D. busckii* or *D. virilis* with *D. melanogaster*. The extension is 500 bp in both 5′ and 3′ direction. Overlap and −log10(*P*-value) is shown for boundaries of TADs (*n* = 2209, 2134, and 2127 in *D. melanogaster*, *D. busckii*, and *D. virilis*, respectively) and SB breakpoints (*n* = 3726 and 3252 in the *D. melanogaster* vs. *D. virilis* comparison, respectively, and 3340 and 2776 in the *D. melanogaster* vs. *D. busckii* comparison, respectively). Overlap with respective shuffled TADs, shuffled SBs, and both TADs and SBs shuffled as controls. A summary of significant and not significant −log10(*P*-value) of Fisher's two-tailed test is shown, the exact *P*-values are provided in Supplemental Table S3. Scheme illustrating the performed overlap analysis. (*B*) Jaccard similarity index of fused TADs and SBs or respective number of shuffled SBs for *D. busckii* and *D. virilis* compared with *D. melanogaster*. For calculating the Jaccard score, consecutive TADs were fused if a SB overlapped the adjacent TAD by 20% or more (see Materials and Methods). The shuffling of SBs is the same as in *A*. The median of called SBs is shown as a green dotted line. *P-*values of two-sided Wilcoxon rank-sum tests are displayed. Scheme illustrating the calculation of the Jaccard similarity index. (*C*) Bitscores of interspecies TAD alignments using BLASTn. TAD to TAD comparisons are displayed in green, TADs shuffled in *D. melanogaster* in light gray, and TADs shuffled in both species in dark gray. Shuffling is the same as in *A*. The median of called TADs is displayed as a green dotted line, and significance was calculated by two-sided Wilcoxon rank-sum test comparisons between the bitscore distributions. All *P*-values are displayed and significant by using a 0.05 *P*-value threshold. Scheme illustrating the BLASTn strategy of whole TADs between two species and the associated bitscore of the best hit.

When visualizing SBs together with genome topology including TADs, TAD boundaries, BUSCOs, and genes (Fig. 2B), we noticed that many SB breakpoints overlap with TAD boundaries. Some corresponding SBs also show maintained TAD architecture (see examples in Fig. 2B; Supplemental Fig. S5E,F). We therefore quantified the significance of this correlation between genomic rearrangements (i.e., SB breakpoints) and the 3D architecture on a genome-wide level, and for this we applied three different methods to validate our findings. First, we computed the overlap of TAD boundaries with SB start and end sites and tested the significance of overlaps while we used the respective shuffled regions in equivalent analyses as controls (see Materials and Methods). This analysis revealed that the overlaps of TAD boundaries and SB breakpoints in all comparisons (Fig. 3A) are highly significant (Fisher's two-tailed *P-*value <2.3 × 10^−62^), whereas for shuffled regions the overlap is decreased to ∼6% and not significant (Fisher's two-tailed *P-*value>0.059, exact *P*-values see Supplemental Table S3). To confirm this result with a second independent analysis method, we investigated to which extent TADs and SBs overlap in length. To do so, we computed the Jaccard similarity index of TADs and SBs or randomly shuffled SBs as a control. This analysis confirmed a significant difference between the overlap of TADs and SBs in comparison with TADs and randomly shuffled regions (see *P-*values of two-sided Wilcoxon rank-sum test in Fig. 3B). As a third approach, we performed pairwise DNA sequence alignments of TADs in the three species and compared their alignment scores with randomly shuffled TADs (Fig. 3C). We surmised that if TADs maintain their integrity during chromosomal rearrangements, their scores obtained from BLASTn (Altschul et al. 1990; Camacho et al. 2009) are expected to be higher compared with random regions. Indeed, we found that the alignment scores (i.e., bitscores) of TADs were significantly higher compared with all controls. This independent sequence-based analysis, which is not taking SBs into account, provided further support for our conclusion that genomic rearrangements in *Drosophila* do not occur randomly, but maintain conserved TADs as units.

### Conserved TADs in *Drosophila* are gene-dense and enriched in histone modifications associated with active transcription

**Figure 4.**
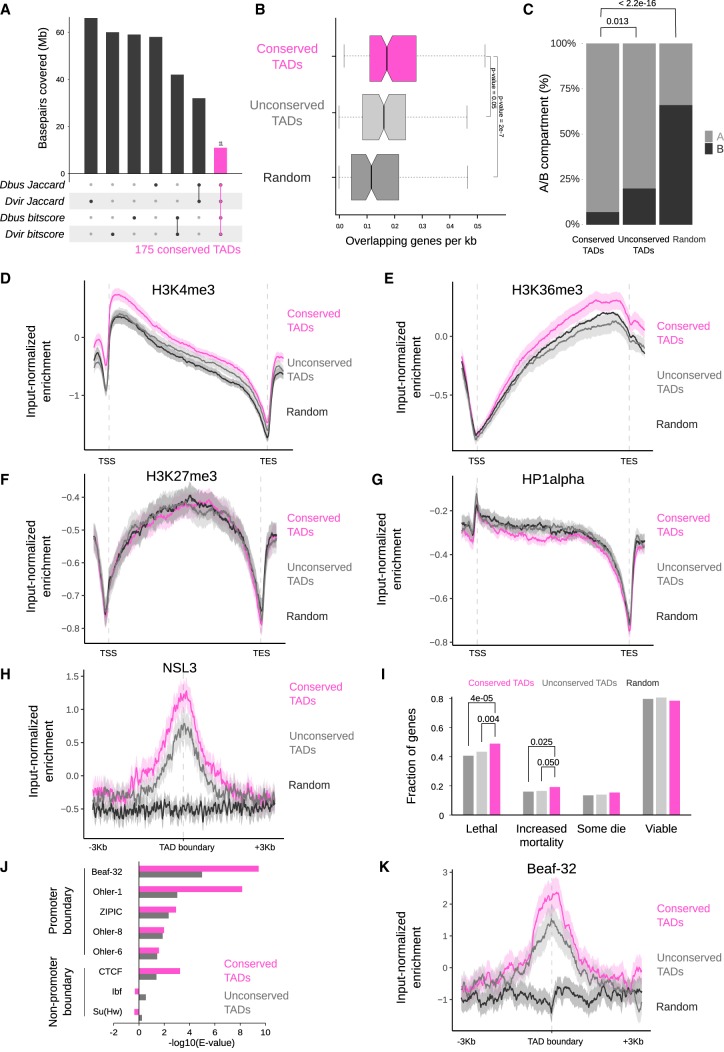
Evolutionary conserved TADs are active gene-rich regions comprising essential genes and are demarcated by conserved boundary motifs. (*A*) Definition of conserved TADs between *D. busckii*, *D. virilis*, and *D. melanogaster.* TADs with Jaccard similarity index above the median from *D. melanogaster* versus *D. busckii* and *D. melanogaster* versus *D. virilis* comparisons were overlapped. Respective overlap of TADs were performed using the bitscores. Afterward, TADs found in both analyses were compared and the intersect was defined as conserved TADs. Barplots represent the base-pair coverage of each subset in the *D. melanogaster* genome. (*B*) Conserved TADs are gene-dense. Genes overlapping with conserved TADs (pink), unconserved TADs (gray), and random genomic regions (dark gray) expressed in number of genes per kilobase. Equal length of overlapping genes is displayed in Supplemental Figure S6B as a control. Wilcoxon rank-sum test *P*-values are displayed for comparisons with conserved TADs. (*C*) Percentage of conserved TADs, unconserved TADs, and random regions that lie completely in the active (A) or inactive (B) compartment (*n* = 101, 101, 92). *P*-values were obtained using a two-sided two-proportions *z*-test. (*D*–*G*) Conserved TADs compared to unconserved TADs are significantly enriched in the H3K4me3 (*D*) and H3K36me3 histone marks (*E*), but are not enriched in H3K27me3 (*F*) or HP1α (*G*). ChIP-seq profiles are from 14- to 16-h old *D. melanogaster* embryos (Celniker et al. 2009). Log2ratio of H3K4me3, H3K36me3, H3K27me3, and HP1α ChIP-seq reads over input reads along genes (transcription start site [TSS] to transcription end site [TES]) in conserved TADs (pink), unconserved TADs (gray), and random regions (black). ChIP-seq profiles show mean (thick line) and 95% CI (shadowed area) of input-normalized ChIP-seq enrichment along scaled genes and unscaled 1 kb before the TSS and after the TES, computed using deepStats (Richard 2019). (*H*) Conserved TADs are enriched in the NSL complex member NSL3. Log2ratio of NSL3 (Lam et al. 2012) ChIP-seq reads over input reads at boundaries of conserved TADs (pink), unconserved TADs (gray), and random regions (black) including the 95% CI (confidence interval) obtained from bootstrapping (*n* = 1000). The NSL3 enrichment at TAD boundaries is significant based on the 95% CI (1.23 ± 0.21 in conserved and 0.78 ± 0.23 in unconserved TAD boundaries). (*I*) Fraction of genes with “lethal,” “increased mortality,” “some die,” or “viable” phenotypic classes defined in FlyBase automatic summaries (genes can be annotated with several phenotypes, see Materials and Methods). Significant *P*-values (*a* = 0.05) for genes intersecting conserved TADs are displayed. They were obtained using one-tailed χ^2^ test to check for proportion differences in two samples. (*J*) Enrichment analysis of promoter and nonpromoter boundary motifs at the boundaries of conserved TADs and unconserved TADs in *D. melanogaster.* Beaf-32 shows the highest motif enrichment at conserved TADs in all three species (see Supplemental Fig. S6G). (*K*) Conserved TADs show higher enrichment of Beaf-32 at their boundaries than unconserved TADs by input-normalized ChIP-seq reads (Van Bortle et al. 2014).

Next, we were interested in elucidating whether conserved genome topology (i.e., TADs) relates to particular gene properties, chromatin states, or functions. We used a stringent definition to identify conserved TADs among all three species by overlapping TADs with high Jaccard similarity indices and BLASTn bitscores (see Materials and Methods and Fig. 4A). We identified 175 conserved TADs corresponding to ∼10% of all TADs, which we compared to an equal number of control TADs (unconserved TADs, see Materials and Methods) or random genomic regions. Conserved TADs appear significantly bigger compared to all TADs (Supplemental Fig. S6A) and more gene-dense (Fig. 4B) compared to unconserved TADs or random regions. The overall length of genes within conserved TADs is similar compared to the other test sets (Supplemental Fig. S6B). A total of 93% of conserved TADs lie within the active A compartment (Fig. 4C), which is a significantly higher proportion compared to unconserved TADs (two-sided two-proportions *z*-test *P*-value = 0.013) or random regions (*P*-value <2.2 × 10^−16^). We next wanted to test whether conserved TADs are enriched for a particular chromatin state and analyzed them for the five chromatin “colors” reflecting active (yellow and red) and inactive (blue, green, and black) states (Filion et al. 2010). Conserved TADs are enriched in the yellow chromatin state associated with broadly expressed genes and the H3K36me3 mark (Supplemental Fig. S6C). As these chromatin “colors” were derived from data obtained in tissue culture cells, we verified this with in vivo data sets from fly embryos and larvae. This confirmed that active chromatin marks, such as H3K4me3 and H3K36me3 (embryos, Celniker et al. 2009) and H4K16ac (third-instar larvae, this study) are significantly enriched (1000 bootstraps 95% CI [confidence interval]) on genes within conserved TADs in comparison with unconserved TADs (Fig. 4D,E; Supplemental Fig. S6D).

On the other hand, marks as H3K27me3 or the heterochromatin protein 1α (HP1α) associated with gene silencing did not show this trend. Given this association with broadly expressed housekeeping genes (Filion et al. 2010), we checked for the presence of known housekeeping regulators such as the NSL complex (Lam et al. 2012) and indeed find NSL3 ChIP-seq enrichment at conserved TAD boundaries (Fig. 4H, bootstrap 95% CI [1.23 ± 0.21 in conserved and 0.78 ± 0.23 in unconserved TAD boundaries]). Functional analyses of genes within conserved TADs showed a modest but significant enrichment (χ^2^ test, *P*-value ≤ 0.05) of genes associated with lethal and increased mortality phenotypes upon mutation, which is in line with housekeeping genes encoding for the most fundamental and universal cellular processes (Fig. 4I; Dickerson et al. 2011). We additionally confirmed a subset of these features of conserved TADs in a set of conserved TADs defined only using the Jaccard similarity index (Supplemental Fig. S6E).

We next turned our attention to the boundaries of these conserved TADs and analyzed them for boundary motif enrichments. In *D. melanogaster*, several DNA-binding proteins are associated with TAD boundaries, for example, the boundary element associated factor-32 (Beaf-32), the motif-1-binding protein (M1BP), the CCCTC-binding factor (CTCF) protein, or suppressor of hairy-wing (Su[Hw]) (Sexton et al. 2012; Van Bortle et al. 2014; Hug et al. 2017; Ramírez et al. 2018). We previously showed that DNA motifs bound by such factors can be used to predict TAD boundaries in *D. melanogaster* (Ramírez et al. 2018) at high resolution. Therefore, we analyzed whether the enrichment of described TAD boundary motifs is conserved in the *Drosophila* species studied here and performed motif enrichment analysis at TAD boundaries in all three species. We focused on motifs previously described in *D. melanogaster* (Ramírez et al. 2018) (see Materials and Methods) to identify their enrichment at TAD boundaries. We find similar enrichments and comparable *E*-values for the same boundary motifs in all three species (Supplemental Fig. S6F). We focused more specifically on Beaf-32, as it displayed the lowest *E*-value at conserved TAD boundaries in all three species (Fig. 4J; Supplemental Fig. S6G). Indeed, enrichment of Beaf-32 assessed by ChIP-seq showed higher enrichment at boundaries of conserved TADs in *D. melanogaster* (Fig. 4K).

Taken together, our results indicate that both conserved and unconserved TADs in all three species maintain conserved boundary motifs. Despite extensive genomic rearrangements, we find that conserved TADs are more active than unconserved TADs with higher A compartment association, active histone marks enrichment, and gene density.

### Spatial contacts between high-affinity sites of the dosage compensation complex are conserved during *Drosophila* evolution

**Figure 5.**
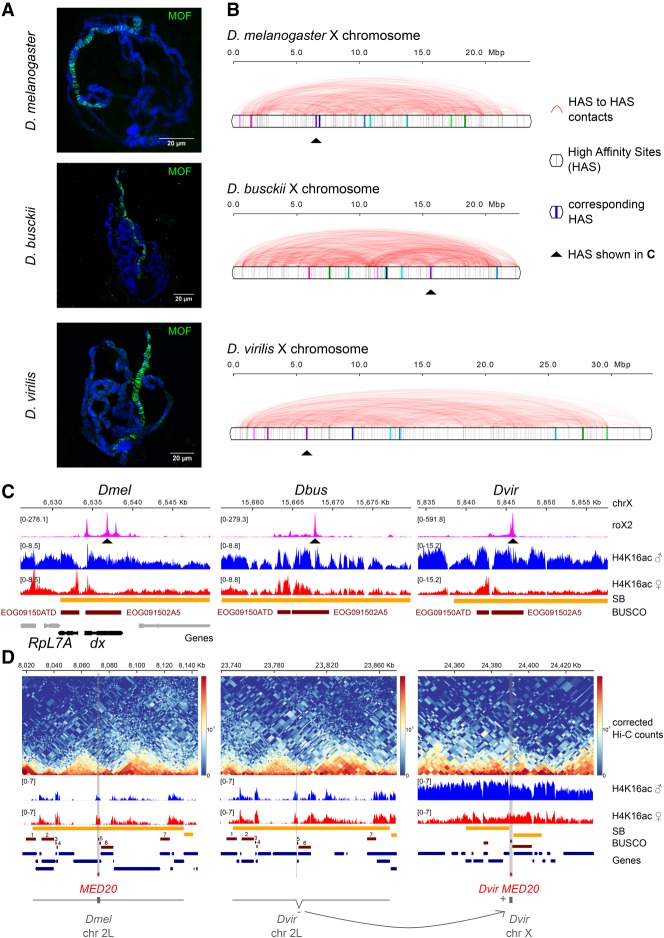
Binding sites of the dosage compensation complex are shuffled along the X chromosome in *D. melanogaster*, *D. busckii*, and *D. virilis*. (*A*) Immunostaining of male polytene chromosomes with MOF antibody (green) in *D. melanogaster*, *D. busckii*, and *D. virilis*. DNA is counterstained with Hoechst (blue). Scale bars, 20 µm. Immunostaining of female polytene chromosomes are shown in Supplemental Figure S7. (*B*) Position (gray vertical bars) and obs/exp Hi-C contacts (red arcs) between high-confidence roX2 sites (HAS) along the entire X chromosome in *D. melanogaster*, *D.* busckii, and *D. virilis*. (*C*) Example of one corresponding HAS as indicated by the red arrows in *B*. Coverage of roX2 ChIRP-seq reads, H4K16ac ChIP-seq reads from separated female and male third-instar larvae, SBs, BUSCOs, and genes annotated in *D. melanogaster*, highlighting *RpL7A* and *dx*, two genes with phenotypic classes related to viability reduction corresponding, respectively, to the BUSCOs EOG09150ATD and EOG091502A5 in all three species. (*D*) Example gene that moved between an autosome and the X chromosome when comparing *D. melanogaster* and *D. virilis*. *MED20* is localized on chromosome 2L in *D. melanogaster* but on chromosome X in *D. virilis* (*Dvir* GJ18844). The surrounding genes on this SB on chromosome 2L maintained the same order (see corresponding BUSCOs numbered from 1 to 7 and gene track). *MED20* in *D. virilis* (*Dvir* GJ18844) is localized on chromosome X in between two surrounding SBs, within a H4K16ac domain (male). Two additional examples are shown in Supplemental Figure S7B,C.

The notion that chromosome conformation hierarchies are maintained during evolution points toward an importance of such structures as entities. X chromosome dosage compensation is one example in which adoption of a specialized chromosome architecture has been functionally associated with its chromosome-wide regulation from worms to mammals (Nora et al. 2012; Crane et al. 2015; Ramírez et al. 2015). In flies, this essential process is orchestrated by the MSL complex, which is composed of the noncoding RNAs roX1 and/or roX2, as well as the proteins MSL1, MSL2, MSL3, MLE, and MOF (Kuroda et al. 2016; Samata and Akhtar 2018). In agreement with earlier findings (Meisel et al. 2012; Quinn et al. 2016), immunostainings of male and female polytene chromosomes showed a strong male-specific enrichment of MOF on the X chromosome of *D. melanogaster*, *D. busckii*, and *D. virilis* (Fig. 5A; Supplemental Fig. S7A). MSL recruitment to the X chromosome occurs at special binding sites termed high-affinity sites (HAS), which are particularly enriched for MSL2, MLE, and roX1/2 and cluster together in space (Alekseyenko et al. 2008; Straub et al. 2013; Ramírez et al. 2015; Schauer et al. 2017; Valsecchi et al. 2018). The presence of HAS sequences and their enrichment of roX appears to be a conserved feature of the X within the *Drosophila* genus (Alekseyenko et al. 2013; Ellison and Bachtrog 2013; Quinn et al. 2016). Given our finding of extensive genome rearrangements, we were interested in how shuffling of the X chromosome impacts dosage compensation and in particular the clustering of HAS into a “dosage compensation hub” in 3D. We defined a comparable set of high-confidence HAS in all three species using roX2 ChIRP-seq data (Quinn et al. 2016) and then analyzed our Hi-C data for the conservation of genome topology at those sites.

By using overlapping BUSCOs, we identified matching HAS by conservation in the three species, which revealed that they have substantially changed their relative position within the X chromosome (Fig. 5B,C). Our chromosome-length assemblies also allowed us to specifically inspect genes that switched between autosomes and the X chromosome in between species. For example, *MED20* moved from chromosome 2L in *D. melanogaster* to the X chromosome in *D. virilis*, where it now resides within a H4K16ac-positive domain (Fig. 5D). Another example of a gene that moved in the opposite direction, namely, from the X chromosome in *D. melanogaster* to chromosome 2L in *D. virilis*, is *mei-41* (Supplemental Fig. S7B). This gene resides in a H4K16ac-positive domain on the X chromosome and only retains a H4K16ac promoter-peak when moved to the autosome. Another example of a gene that moved from chromosome 2L in *D. melanogaster* to the X chromosome in *D. virilis* and there gained a species-specific HAS is the gene *dim gamma-tubulin 3* (Supplemental Fig. S7C).

**Figure 6.**
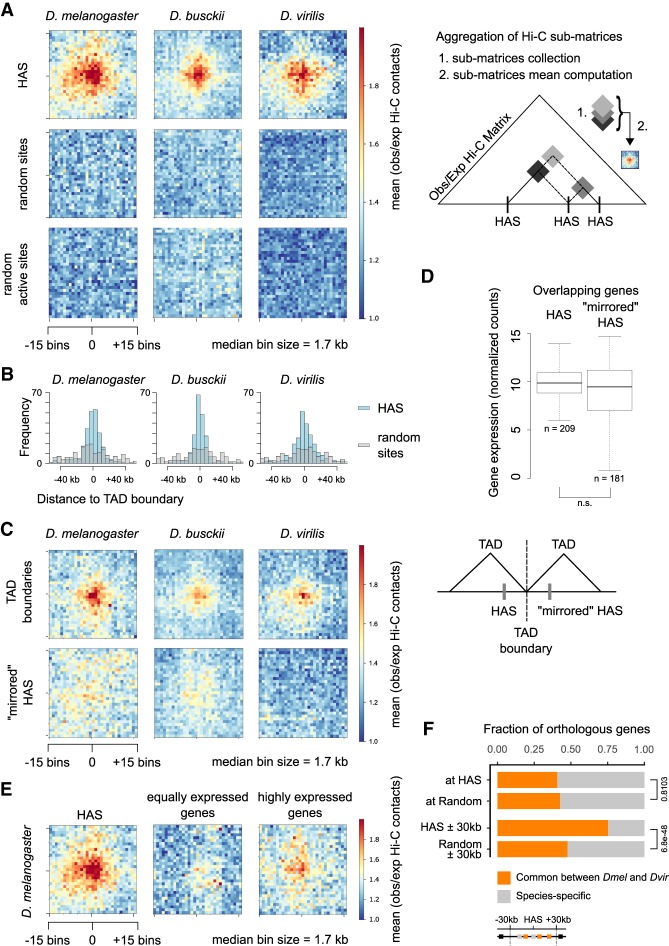
Enriched Hi-C contacts between binding sites of the dosage compensation complex are maintained throughout *Drosophila* evolution despite genome shuffling. (*A*) Aggregate Hi-C matrices around pairwise HAS–HAS contacts in *D. melanogaster*, *D. busckii*, and *D. virilis* compared to random and random active (sites in the A compartment within SBs) pairwise contacts. Displayed are the mean observed over expected contacts ratios of corrected Hi-C matrices with an ∼1.7-kb bin size of ∼250 HAS that are on the X chromosome (*n* = 246, 213, and 247 in *D. melanogaster*, *D. busckii*, and *D. virilis*, respectively) or a respective number of random regions on the X chromosome. Scheme illustrating the generation of aggregate Hi-C matrices (aggregate plots). (*B*) Distance of HAS used in *A* to closest TAD boundary compared to the respective number of random sites. (*C*) Aggregate Hi-C matrices centered on TAD boundaries with the lowest insulation score on the X chromosome in *D. melanogaster*, *D. busckii*, and *D. virilis* (*n* = 246, 213 and 247 in *D. melanogaster*, *D. busckii*, and *D. virilis*, respectively), and HAS mirrored at their closest TAD boundary (“mirrored” HAS). TAD boundaries show enriched contacts but “mirrored” HAS show no enriched contacts. (*D*) Gene expression (normalized counts) of genes overlapping with HAS compared to “mirrored” HAS is not significantly different (n.s.) by Wilcoxon rank-sum test. Comparison of gene expression was performed using library-size normalized RNA-seq counts from 14- to 20-h aged embryos from modENCODE data sets obtained from Ramírez et al. (2018) and also available on the Chorogenome web server (http://chorogenome.ie-freiburg.mpg.de/). (*E*) Aggregate Hi-C matrices around HAS–HAS or TSS–TSS contacts of genes with equally high expression as genes overlapping with HAS or highly expressed genes in *D. melanogaster* (*n* = 209). Underlying gene expression values are shown in Supplemental Figure S7D. (*F*) Fraction of total orthologous genes (gray) and orthologous genes in common between *D. melanogaster* and *D. busckii* (orange) at HAS (*n* = 195), at random positions defined in the X chromosome A (active) compartment (*n* = 175), at HAS extended by 30 kb in 5′ and 3′ direction (*n* = 1380), and at 60-kb random regions defined in the X chromosome A (active) compartment (*n* = 838). Reported values are calculated in *D. melanogaster*. Two-sided two-proportions *z*-test *P*-values are shown on the *right* of the bar plot. Orthologs between *D. melanogaster* and *D. virilis* were retrieved from FlyBase.

In light of the genomic rearrangements occurring in the three species, we asked whether spatial contacts between HAS could possibly be conserved. For this, we generated aggregate plots (aggregate Hi-C matrices) for quantifying the mean of aggregated/stacked Hi-C submatrices between HAS pairs after an obs/exp transformation of the Hi-C matrix. This revealed an enrichment of Hi-C contacts for HAS, but not random regions, irrespective of whether those were sampled from the entire X chromosome or only from active regions (Fig. 6A). This highlights the maintenance of HAS interactions despite extensive shuffling of the genomes.

HAS are enriched near TAD boundaries in *D. melanogaster* (Ramírez et al. 2015). We wanted to analyze whether this also holds true in other *Drosophila* species and calculated the distance of the 250 high-confidence HAS to the closest TAD boundary (Fig. 6B). Indeed, *D. virilis* and *D. busckii* HAS, but not random regions, are also enriched in the vicinity of TAD boundaries. In agreement with earlier observations in *D. melanogaster* (Hug et al. 2017), we were also able to generally identify enriched TAD boundary contacts in *D. busckii* and *D. virilis* (Fig. 6C). Because HAS tend to be in the proximity of boundaries, we next wanted to test whether HAS–HAS contacts were simply driven by their “closeness” to boundaries. For this, we defined a set of alternative, non-HAS genomic positions around TAD boundaries (defined as “mirrored” HAS), which have the same distance as HAS to TAD boundaries but are located on the opposite site of the closest TAD boundary. Interestingly, we do not find enriched contacts at these “mirrored” HAS (Fig. 6C). Importantly, gene expression levels between HAS genes (*n* = 209) and mirrored HAS genes (*n* = 181) were similar (Fig. 6D). Furthermore, non-HAS genes, which are expressed equally strong or higher than HAS genes, showed lower Hi-C contacts in comparison with HAS (Fig. 6E; Supplemental Fig. S7D). This suggests that enriched contacts between HAS are neither a mere consequence of closeness to TAD boundaries nor the expression level of their associated genes.

We next inspected the genes at HAS between *D. melanogaster* and *D. virilis* and for this used genes in the A compartment as controls, because HAS are mostly found within this compartment. Using HAS positions in the *D. melanogaster* genome, comparison of orthologs showed that about 40% of genes at HAS are in common between the two species (Fig. 6F). This fraction is similar to what would be expected by chance in the A compartment (*P*-value = 0.81), as around 42% of the genes are common orthologs between *D. melanogaster* and *D. virilis*. We next turned our attention to genes that surround HAS. As of HAS spacing of around 60 kb in all three species and the extent of MSL spreading from HAS (Kim et al. 2018) we inspected genes within a window of 30 kb around HAS. Indeed, when comparing these genes in the vicinity of HAS (±30 kb), we find ∼73% conserved orthologous genes, which is significantly different from active random regions showing 46% conserved orthologous genes (*P*-value < 2.2 × 10^−16^). The respective analysis in *D. virilis* showed similar trends (*P*-value = 2.3 × 10^−4^ at HAS and 6.2 × 10^−21^ ± 30 kb around HAS). Because HAS themselves are not enriched at genes that are evolutionary conserved, whereas the genes in their vicinity (30 kb) are, we asked whether individual conserved genes in the vicinity of HAS rely on the same HAS for compensation or not. To test this, we determined each HAS identity between *D. melanogaster* and *D. virilis* by finding pairs of HAS with the highest fraction of common orthologous genes between both species compared to all orthologous genes in their vicinity (±30 kb) (Supplemental Fig. S7E). We repeated this analysis for 100 sets of random 60-kb regions in *D. melanogaster* and *D. virilis* and report no significant difference from extended HAS (Wilcoxon test *P*-value = 0.99 after adjustment for multiple comparison by Benjamini–Hochberg procedure). This suggests that compensated genes stay in the vicinity of any HAS but not necessarily the same HAS over evolution. GO term analysis of genes around HAS showed similar enriched functions compared to respective random regions (Supplemental Fig. S7F). This suggests that compensated genes are evolutionary conserved and involved in diverse and essential biological functions.

Taken together, our data suggest that regulatory relevant genome 3D architecture (e.g., HAS contacts involved in X chromosome dosage compensation) can be maintained during evolution independently of chromosome rearrangements.

## Discussion

Here, we analyzed the impact of genomic rearrangements on genome topology using the *Drosophila* genus and X chromosome dosage compensation as a model system. After generating Hi-C-assisted chromosome-length assemblies of the *D. busckii* and *D. virilis* genomes, we identified and characterized conserved TADs as evolutionary maintained units in three *Drosophila* species and further analyzed the conserved spatial network of HAS contacts on the X chromosome.

Hi-C scaffolding can produce high-quality genome assemblies (Burton et al. 2013; Kaplan and Dekker 2013; Korbel and Lee 2013; Marie-Nelly et al. 2014; Bickhart et al. 2017; Dudchenko et al. 2017; Mascher et al. 2017). In our study, we generated chromosome-length genome assemblies by combining Hi-C data with either de novo assembled contigs of homogeneous size (in the case of *D. busckii*) or published scaffolds of diverse sizes (in the case of *D. virilis*). One advantage of integrating Hi-C data in assembly projects is that it provides high-quality data at low cost: Our Hi-C libraries for scaffolding were sequenced at a coverage of as little as 17× for *D. busckii* and 19× for *D. virilis.* By comparison, Chakraborty et al. used 121× PacBio read coverage to produce chromosome-length scaffolds for *D. melanogaster* (Chakraborty et al. 2016, 2018). Combining these two technologies provides a powerful approach, because even with long-read sequencing alone, stretches of repetitive regions can be too long to be spanned, resulting in fragments (scaffolds) with unknown order, orientation, and assignment to a chromosome. On the other hand, both Hi-C-assisted assemblies in our study still contain a small fraction of unplaced, mostly repetitive or tiny fragments, which is a common challenge. Long-read sequencing can aid in the assembly of such repetitive regions, and accordingly, we note that the fraction of unplaced fragments in our *D. busckii* genome assembly is lower compared to the one of *D. virilis.* Although the analyses discussed hereafter do not integrate repetitive regions and are thus not affected by their omission, it will be important to further improve this for studying genome evolution of repeats. Nevertheless, our two chromosome-length genome assemblies of *D. virilis* and *D. busckii* show an unprecedented continuity compared to the previous assemblies of these species’ genomes and thus constitute a valuable resource for further genetic and evolutionary studies in *Drosophila*.

Apart from its advantages for genome assembly, Hi-C additionally provides information of chromosome architecture. Thus, we used this data to comprehensively define and characterize conserved sequences between *D. melanogaster*, *D. busckii*, and *D. virilis.* Genome alignments revealed that genomic rearrangements during evolution occurred mostly within the entire length of the same chromosome arms (Fig. 2A). This observation is in accordance with previous reports in *Drosophila* (Drosophila 12 Genomes Consortium et al. 2007) and the mosquito species *Aedes aegypti*, *Culex quinquefasciatus*, and *Anopheles gambiae*, which are separated by about 150 to 200 million years of evolution (Dudchenko et al. 2017). Moreover, we identified synteny blocks (SBs) between the three *Drosophila* species and found conservation of active and inactive (A/B) compartments in these regions and report significant overlap of synteny breakpoints with TAD boundaries. Conservation of A/B compartments has to our knowledge only been studied in plants (Dong et al. 2017; Xie et al. 2019). We demonstrate that conserved TADs are mainly found in the active compartment and that genes within conserved TADs are enriched for active histone modifications. Our genome-wide analyses confirm that conserved TADs may indeed explain a fraction of the nonrandom distribution of synteny breakpoints. The occurrence of evolutionary rearrangements at TAD boundaries was also described in a gibbon–human comparison (Lazar et al. 2018). This study identified 67 rearrangements between both species within an evolutionary distance of ∼17 million years. The *Drosophila* species analyzed in our study cover ∼40 million years of evolution and multiple subgenera (Russo et al. 1995; Robe et al. 2010). We observe that *Drosophila* genomes are apparently extremely rearranged with, on average, 20 synteny breakpoints per Mb despite being highly gene dense. This high number of synteny breakpoints is in agreement with earlier reports and may be ascribed to the short generation time of *Drosophila* compared to mammalian species (Bhutkar et al. 2008; Thomas et al. 2010). A recent study confirmed that synteny breakpoints overlap with TAD boundaries in multiple vertebrate species and showed that gene expression of orthologs in conserved TADs is higher compared to orthologs in unconserved TADs (human to mouse comparison) (Krefting et al. 2018). *D. melanogaster* balancer chromosomes that contain eight inversions were used to show that rearrangements that shuffle TADs rarely affect gene expression (Ghavi-Helm et al. 2019). Possibly, different mechanisms are involved in vertebrates versus insects. Reasons for such differences could be that TAD boundaries in flies, in contrast to vertebrates (e.g., mammals or zebrafish), are mostly not demarcated by CTCF, but several DNA-binding proteins are associated with TAD boundaries (Ramírez et al. 2018). Therefore, the comparison of *Drosophila* species that share extremely similar functional constraints, gene content, developmental progression, and life span is extremely powerful in elucidating fundamental properties of conserved TADs despite thousands of genomic rearrangements and allows characterization of their features. Their functional relevance and/or their regulatory surroundings may explain why they have been maintained during evolution. We hypothesize that ancient TADs are conserved because rearrangements are negatively favored during evolution within these domains containing active, gene-dense regions important for maintaining cell integrity. However, factors other than TADs are involved in the determination of synteny breakpoints, as a significant fraction cannot be associated with a conserved TAD structure, at least with our analyses. In mammals, retrotransposition was shown to create new CTCF-binding sites by repeat-element expansions (Schmidt et al. 2012). In principle, such a mechanism could be also operating in flies. Further studies are required to elucidate, if there is any preference of chromosome breakage at certain sequences, e.g., during germ cell formation and how such rearranged chromosomes are inherited and selected against later on during development, in particular if regulatory topology and thus possibly gene expression is affected.

Genome topology is involved in facilitating the spreading of the dosage compensation complex on the X chromosome by spatial proximity of HAS (Ramírez et al. 2015; Schauer et al. 2017). This 3D conformation is maintained during evolution in *Drosophila*, as we observe enriched HAS contacts in all three analyzed species. Taking into consideration that genomes are completely shuffled during evolution, the conservation of dosage compensation (Alekseyenko et al. 2013; Quinn et al. 2016) and the recognition of the X chromosome specifically is indeed remarkable. This suggests that enriched contacts between HAS are conserved in the *Drosophila* genus because of their functional importance for dosage compensation. Indeed, our analyses imply that HAS do not exhibit spatial proximity simply because of their closeness to TAD boundaries or gene activity, but rather intrinsically provide this property. Yet, this seems not to involve male-specific factors, as such enriched contacts are found in both males and females (Ramírez et al. 2015; Schauer et al. 2017). The idea of spatially colocalized binding sites was suggested for transcription factors and enhancers. Three-dimensional proximity can facilitate diffusion and increase local concentration of factors along the genome (Brackley et al. 2012; Liu et al. 2014; Malin et al. 2015; Pernuš and Langowski 2015; Ma et al. 2018). Further studies are required to understand the molecular mechanism of 3D interaction site formation of HAS on the X chromosome. Another hint toward the importance of spreading but not necessarily the location of individual HAS themselves is that genes in the vicinity of HAS are more conserved than genes overlapping with HAS. This suggests that the spatial proximity of HAS and the spreading of the MSL complex from them is more critical with respect to the compensation function than the absolute positioning and order of individual HAS. Thus, HAS seem interchangeable and contributing equivalently to dosage compensation across evolution. This could be similar for other binding sites that display spatial proximity and are important for essential regulatory mechanisms, for example enhancers. Further studies are required to test this hypothesis.

In summary, the Hi-C-guided *Drosophila* assemblies and the strategies for comparing chromatin conformation data between species presented in our study provide insights into genome topology evolution. In particular, our finding of evolutionary stability of entire regulatory units on chromosomes and topology including a full chromosome, despite genome shuffling, may be an important step toward a further understanding, in how changes and mutations affecting gene topologies may impact on essential cellular processes in all eukaryotic kingdoms.

## Materials and methods

### *D. melanogaster*, *D. virilis*, and *D. busckii* fly lines

The following fly lines were used for experiments: *D. melanogaster* (*w*^*1118*^ Oregon R *Drosophila melanogaste*r), *D. virilis* (*Drosophila virilis*, San Diego stock center, stock number: 15010-1051.00), and *D. busckii* (*Drosophila busckii*, San Diego stock center, stock number 13000-0081.31).

*D. melanogaster* and *D. virilis* flies were maintained at room temperature on standard cornmeal-molasses medium. *D. busckii* flies were additionally fed with instant *Drosophila* medium (Formula 4–24, Carolina Biological Supply Company, catalog number 173202) mixed with instant potato powder on top of the standard *Drosophila* medium.

### Fly embryo collection and fixation for in situ Hi-C

Flies were transferred into collection cages at least 1 d before embryo collection at 25°C. Pre-lays were done for 2 h and fly embryos were collected on apple juice plates with yeast for 2 h and then aged at 25°C until they reached developmental stage 15–16. Because embryogenesis timing differs across species (Kuntz and Eisen 2014) we collected 16- to 18-h-old *D. melanogaster*, 21- to 23-h-old *D. virilis*, and 19- to –21-h-old *D. busckii* embryos (see Supplemental Fig. S1). Hi-C data from 21- to 23-h-old *D. busckii* embryos was used for the genome assembly of *D. busckii*. Because no differences in the 3D chromatin conformation were found between males and females, we used mixed embryos in our experiments (Ramírez et al. 2015; Schauer et al. 2017).

*D. melanogaster* and *D.virilis* embryos were dechorionated, washed, and fixed for 15 min in 5 mL of 1% methanol-free formaldehyde in PBS with 5 mL heptane while shaking. Fixation was stopped by adding glycine up to a final concentration of 0.25 M and incubating for 5 min. The fixation solution was removed and embryos were washed twice in 0.1% Triton-X in PBS for 10 min. Supernatant was removed and samples stored at −80°C. To maintain the integrity of all nuclei, including those far from the embryo surface, we fixed *D. melanogaster* and *D. virilis* embryos again while breaking them into smaller fragments in a 1-mL dounce homogenizer using 1% methanol-free formaldehyde in serum-free Schneider's medium supplemented with 0.5% NP-40 for 10 min at room temperature. Fixation was quenched by adding glycine up to a final concentration of 0.125 M and immediate pelleting of fly embryo fragments at 1000*g* for 5 min. Samples were washed in PBS and then kept on ice for nuclei extraction (see next paragraph). *D. busckii* embryos are smaller than embryos from the other two fly species (Gregor et al. 2005). Fixation using the above described procedure led to loss of many embryos during fixation. Therefore, *D. busckii* fly embryos were dechorionated and directly fixed while breaking them into smaller fragments in a 1-mL dounce homogenizer as described above.

### In situ Hi-C of *D. melanogaster*, *D. virilis*, and *D. busckii* embryos

In situ Hi-C experiments were performed using a modified version of the in situ Hi-C protocol (Rao et al. 2014) described in Ramírez et al. (2018). Nuclei were extracted by resuspension in 1 mL ice-cold lysis buffer (10 mM Tris-HCl, pH 8, 10 mM NaCl, 0.2% IGEPAL CA-630) and sonication following the standardized nuclear extraction NEXON protocol (Arrigoni et al. 2016) (Covaris E220 sonicator, settings: 75 W peak power, 2% duty factor, 200 cycles/burst, for 15–30 sec until ∼70% of intact nuclei were released). Samples were filtered through a 30-µm filter to remove bigger embryo fragments. From this step on we followed the protocol described in Ramírez et al. (2018). Nuclei were digested using DpnII (NEB, R0543M, 150 units/sample). To increase the fraction of valid Hi-C reads (see Supplemental Fig. S1), dangling ends were removed after purification of Hi-C ligated DNA in samples from *D. busckii* using 5 units of T4 DNA polymerase for 30 min at 20°C with addition of 25 µM dGTP. After biotin pull-down, 50 ng of DNA bound to beads was used for library preparation and libraries were sequenced paired-end with a read length of 75 bp, on a Illumina HiSeq 3000 or Illumina NextSeq500 machine. Supplemental Table S2 provides the numbers of sequenced and filtered valid reads of all Hi-C samples.

### De novo *D. busckii* hybrid contig assembly

To assemble the *D. busckii* genome we followed a hybrid approach (Fig. 1A) in which we combined short Illumina and long PacBio reads. Illumina reads of female flies were obtained from Vicoso and Bachtrog (2015) and Zhou and Bachtrog (2015) accession codes SRR1795010, SRR1794619, SRR1794616, SRR1794617, SRR1794614, and SRR826809. These paired-end reads of whole-genome sequencing data were trimmed for adapters and sequencing quality using Trim Galore (v0.4.0). All reads (68 bp average length, 18.8 Gb, 156× coverage) were merged like single-end data and assembled into contigs using SparseAssembler v20160205 (Ye et al. 2012) with parameters “*k 51 g 15 NodeCovTh 1 EdgeCovTh 0 TrimN 2 GS 240000000*.” We did not include the paired-end information from Illumina reads using SparseAssembler to reduce errors introduced by heuristics usually applied in short-read assembly as gap closing or scaffolding. Short-read assembly resulted in 32,010 contigs with N50 of 8.9 kb.

PacBio reads of genomic DNA were generated by GATC Biotech from 100 adult female *D. busckii* flies. PacBio reads were sequenced on a RS II system using P6 chemistry. In total, 2.7 Gb PacBio data (20× coverage) were obtained with a mean polymerase read length of 8.2 kb and a mean subread length of 5.7 kb. The FM-index Long Read Corrector FMLRC (Wang et al. 2018) was used to reduce sequencing errors of the PacBio reads by using short Illumina reads.

In a second assembly step, PacBio reads were integrated by aligning and overlapping the previously generated high-confidence contigs to the much longer but error-prone PacBio reads using DBG2OLC v20160205 (Ye et al. 2016) with parameters “*LD 0 k 17 AdaptiveTh 0.01 KmerCov 2 MinOverlap 20 RemoveChimera 1 ChimeraTh 2*.” DBG2OLC assembly resulted in a total assembly length of 120,159,444 bp consisting of 245 contigs with N50 of 1,441,251 bp (Table 1). This hybrid assembly approach takes advantage of highly accurate Illumina reads and long third-generation sequencing reads. The next paragraph describes chromosome-length scaffolding of these contigs using Hi-C data.

We have tested several additional assembly methods for contig assembly that allow combining PacBio and Illumina reads or PacBio data alone, namely, Canu (Koren et al. 2016), Miniasm (Li 2016), Spades (Bankevich et al. 2012), and Masurca (Zimin et al. 2013). After Hi-C scaffolding, we evaluated the total assembly length, mapping rate of Hi-C data to the assemblies, and frequency of obvious misassemblies by visual inspection of the automatically generate Hi-C matrices from HiCAssembler. We concluded that the assembly provided by SparseAssembler in combination with DBG2OLC was the best and generated subsequent assemblies by fine tuning the parameters to reduce misassemblies that are clearly visible as part of the assembly of the contigs using Hi-C.

### Hi-C assembly algorithm

To assemble the *D. busckii* and *D. virilis* genomes we used an iterative scaffolding strategy similar to 3D-DNA (Dudchenko et al. 2017). To determine the order of scaffolds we used a maximum spanning tree as in LACHESIS (Korbel and Lee 2013). Our algorithm is open source, easy to install, use, and is freely available at https://github.com/maxplanck-ie/HiCAssembler.

Our assembly of chromosome-length Hi-C scaffolds can be done using preassembled short contigs or scaffolds and consists of the following steps (in this Materials and Methods section we will use “scaffolds” to refer to preassembled short contigs or already available scaffolds):

#### Creation of corrected Hi-C contact matrix

Read mapping: Reads are aligned to the preassembled scaffolds, each mate is aligned separately using BWA MEM (Li 2013) with parameters -A1 -B4 -E50 -L0 (which promote a read to be split instead of adding a gap). Creation of Hi-C contact matrix: “*hicBuildMatrix*” from HiCExplorer (Ramírez et al. 2018) is used to compute the Hi-C contact matrix after filtering out low-quality reads. They consist of reads that are mapping to several repetitive regions, that are not close to restriction sites, that did not religate (dangling ends), self-circles and same-fragment reads. Information about read filtering during matrix creation is provided in a quality control (QC) summary of each Hi-C sample. Bin size is set to restriction fragment length. Matrix correction: The total number of reads that are assigned to each bin is calculated. Bins having zero or low number of reads as well as bins having read counts over 1.6 median absolute deviation scores (MAD-score) are removed. The elimination of bins with a low number of reads avoids amplification of signal from these bins during the matrix-balancing correction step. The elimination of bins with a MAD *z*-score of 1.6 or larger aims to reduce bins containing collapsed repetitive regions due to assembly errors. Collapsed repetitive regions refer to genomic repetitions that appear as unique in preassembled scaffolds that can be identified in some cases by a high coverage. After bin filtering, the matrix is corrected using iterative correction (Imakaev et al. 2012) implemented in the “*hicCorrectMatrix*” tool from HiCExplorer.

#### Detection of misassemblies

Misassemblies are a common problem in de novo or published assemblies using Illumina and/or PacBio technologies and it is important to remove them; otherwise they introduce significant errors in Hi-C-based assemblies. Misassemblies are readily spotted as discontinuous regions in Hi-C contact matrices (see Supplemental Fig. S3E) that do not follow the power law decay with respect to genomic distance (Lieberman-Aiden et al. 2009). HiCAssembler provides two methods to remove misassemblies.

##### Automatic method

Most misassemblies can be detected automatically when they occur between scaffolds located in different chromosomes or far away from each other in genomic distance. To detect misassemblies we use the HiCExplorer TAD detection algorithm based on the TAD-separation score. Misassemblies can be identified as positions in the genome in which the adjacent downstream and upstream regions share significantly less contacts compared to the global average. Thus, the problem is similar to that of identifying TADs based on local minima of the TAD-separation score (Supplemental Fig. S3E). When running HiCAssembler, a cutoff of the TAD-separation score can be given to split scaffolds. Erroneously split strong TAD boundaries are put together again during the assembly. Unsplit misassemblies can be detected by visual inspection of the HiCAssembler output that documents the Hi-C assembly process (Supplemental Fig. S3E).

##### Manual method

Some misassemblies cannot be removed automatically, specifically when they are close to their correct genomic location, close to the borders of scaffolds, or in small scaffolds where it is not possible to accurately compute the TAD-separation score. These misassemblies can be detected by visual inspection of the contact matrix. It is possible to instruct HiCAssembler where to add splits using the “–*split_positions_file*” parameter. HiCAssembler integrates a GUI tool called “*plotScaffoldsInteractively*” that allows researchers to look and zoom into any single scaffold and to identify exact genomic positions of desired split points.

#### Creation of initial path graph

To keep track of the Hi-C assembly process, HiCAssembler uses a path graph. In this type of graph, nodes can only be connected to at most two other nodes and cycles are not allowed. HiCAssembler creates a path graph in which each node is a bin of the corrected Hi-C contact matrix and scaffolds are represented by paths connecting their corresponding nodes. Once a path is created, new connections are only allowed when involving the first or last node.

HiCAssembler internally maintains two path graphs. Apart from the path graph joining bins of the Hi-C contact matrix, a second graph is constructed in which scaffolds are nodes (Supplemental Fig. S3B) and, as the Hi-C assembly progresses, paths of scaffolds are created in sync with larger paths connecting their corresponding bin paths.

#### Removal of tiny scaffolds and user-defined problematic scaffolds

In the next step, tiny scaffolds are removed from the path graph but they will be reintegrated in the Hi-C assembly at later stages. The length threshold to remove scaffolds is a user-defined parameter in HiCAssembler; however, in our experience we have found that scaffolds of <100 kb tend to introduce errors as they share fewer contacts with other scaffolds and are thus less reliably ordered. Scaffolds containing repetitive regions can introduce ambiguities and should therefore be manually removed during the initial Hi-C assembly steps.

#### Iterative joining of high-confidence scaffold paths

HiCAssembler progresses by iteratively joining scaffolds to form larger and larger paths in each iteration until chromosome-length assemblies are obtained (Supplemental Fig. S3A). The first iteration starts with preassembled contigs or scaffolds. The Hi-C scaffolds output from each iteration are the input for the next iteration. During each iteration the individual steps taken are (1) merging of the initial matrix bins to create a smaller matrix, (2) determination of a confidence cutoff score, (3) transformation of the merged matrix into a graph and computation of the maximum spanning tree, (4) resolution of hubs, and (5) orientation and extension of scaffolds. We will use the term Hi-C scaffold to refer to a joined and oriented set of scaffolds.

1. **Merging initial matrix bins**
  During each iteration, the Hi-C matrix is reduced by merging bins that belong to one Hi-C scaffold (Fig. 1C). The internal bins of each Hi-C scaffold are merged into parts that are about the size of the smallest Hi-C scaffold. Thus, some Hi-C scaffolds’ bins are all merged together into one new bin while other scaffolds may contain several new larger bins (Supplemental Fig. S3B, second panel). Merging bins allows more Hi-C data to be aggregated per bin and increases confidence in the analysis. HiCAssembler uses fast algorithms that efficiently merge matrix bins. Because each new bin is the result of merging a variable number of smaller bins, the new matrix is corrected using an optimized version of the iterative correction method (Imakaev et al. 2012).
2. **Determination of a contact cut-off threshold to keep chromosomes separated**
  To estimate the number of contacts that are shared between scaffolds that are consecutive or separated by the average Hi-C scaffold length, we compute the median number of contacts between all parts of divided Hi-C scaffolds at all distances. These values are used to determine a cut-off threshold that will remove any contact between bins below this threshold. The cut-off threshold is set to the median number of contacts between Hi-C scaffolds that are separated by the length of one Hi-C scaffold. This will avoid joining scaffolds from distinct chromosomes but also avoids joining Hi-C scaffolds that are separated from each other by at least the distance of the smallest Hi-C scaffold in each iteration. This step is different from the strategies used by LACHESIS (Burton et al. 2013) and 3D-DNA (Dudchenko et al. 2017) to differentiate chromosomes: LACHESIS requires an initial clustering of scaffolds into a given number of groups, whereas 3D-DNA uses a misassembly removal algorithm after the assembly process to separate the “mega scaffold” into chromosomes. In our opinion, this strategy makes the Hi-C assembly simpler by avoiding the initial clustering or the final separation of chromosomes.
3. **Transformation of the merged matrix into a graph and computation of the maximum spanning tree**
  The merged contact matrix is transformed into a weighted graph in which each node is a Hi-C scaffold (or a part of a large Hi-C scaffold) and each edge weight is the corrected number of contacts shared by one pair of Hi-C scaffolds in the merged matrix (Supplemental Fig. S3B,C). The cut-off threshold from the previous step is applied to remove all edges whose weight is below the threshold (Supplemental Fig. S3, second panel). A maximum spanning tree (MST) is applied to this graph as in LACHESIS (Supplemental Fig. S3, third panel; Korbel and Lee 2013). The MST algorithm removes any cycles in the graph and leaves only the edges with the highest weight. The graph before the MST algorithm and after the MST is saved in the .graphml format. Those graphs can be visualized using, for example, Cytoscape (Shannon et al. 2003) and can be useful to identify problematic nodes that can afterward be manually removed from the assembly using the “–*scaffolds_to_ignore*” option.
4. **Resolution of hubs**
  After applying the MST, the resulting graph may contain nodes that are connected by more than two other nodes. We refer to these nodes as hubs. During the first iteration (before any scaffold has been attached to others) any branch containing only one node is pruned from the graph. These pruned nodes are put aside and integrated together with the tiny scaffolds after the Hi-C assembly of larger scaffolds finishes. Other hubs are resolved by leaving the top two edges with the highest weight and removing all other edges (Supplemental Fig. S3C, last panel).
5. **Orientation and extension of scaffolds**
  After hub removal, the graph contains only paths in which each node is either a complete Hi-C scaffold or part of a divided large Hi-C scaffold (Supplemental Fig. S3B, last panel). New connections between Hi-C scaffolds are now added. The following steps are carried out to resolve the orientation of scaffolds: (1) Identify all unmerged high-resolution bins that belong to each part of the Hi-C scaffolds (Supplemental Fig. S3D, left). High-resolution bins correspond to bins in the original Hi-C matrix before step 1 of the iteration. (2) A small Hi-C matrix, containing only the selected bins is created. (3) Bins in the matrix are rearranged by keeping all bins that belong to one scaffold either in the same order (forward orientation) or in the inverted order (reverse orientation) (Supplemental Fig. S3D, right). For each possible scaffold orientation, the hic-score is computed using the following equation:
  $$\mathrm{hic}\text{-}\mathrm{score}\;=\sum_{i=1}^{n}\sum_{\;j=i+1}^{n}a_{i,j}(i\;-j).$$

Here, *n* is the size of the small submatrix, and *a*_*i*,*j*_ is the value in the matrix for row *i* and column *j*.

The orientation of scaffolds that results in the matrix that minimizes the hic-score is used to join and orient Hi-C scaffolds. Internally, the path graph of matrix bins and the path graph of scaffolds are updated.

In the hic-score function, values that are away from the main diagonal are multiplied by an increasingly higher number (*i−j*). Thus, only those Hi-C matrices having a high number of contacts close to the main diagonal will have low scores. This is expected because a correct Hi-C conformation is characterized by an approximate exponential decrease in the number of counts when moving away from the main diagonal of the matrix. This can be seen in the submatrices depicted in Supplemental Figure S3D (right).

At the end of each iteration an image of the complete Hi-C matrix is saved. Because the hardware requirements grow quadratically with respect to the matrix size it is not practical to plot a matrix that has more than approximately 4000 bins. Thus, the Hi-C matrix is reduced to at most 4000 bins. These images are useful to detect problems with the assembly and to decide if adjustments such as manual splitting or removal of (erroneous parts of) scaffolds are needed.

#### Incorporation of tiny scaffolds

Once the iterative joining of Hi-C scaffolds ends, tiny scaffolds that were not used yet are put back into the Hi-C assembly. For this, paths of removed scaffolds are identified and inserted next to the Hi-C scaffold node with the highest number of contacts. In detail, first, a cut-off threshold is computed as in step 2, but this time the median of contacts for consecutive bins is used. Then, all scaffold bins are merged to form a smaller matrix as in step 1, the matrix is converted to a graph whose edges have weight = number of corrected contacts if the edge connects a removed scaffold. Otherwise, if the edge connects two Hi-C scaffold nodes already ordered and oriented, the edge weight = max (number of corrected contacts in the graph). In other words, all edges between scaffolds that were already joined in the iterative assembly have a maximum value. Afterward, the cutoff is applied to remove low-scoring edges. Then the MST is computed. Because all edges between Hi-C scaffolds already joined have the maximum value, none of those edges is removed in the MST computation. This creates a graph in which the removed scaffolds either form branches that are attached to a single Hi-C scaffold or are an independent tree. Next, we iterate over each branch and tree; if the branch/tree forms a path, the orientation of its scaffolds is determined as in step 5. If the branch is connected to a Hi-C scaffold, the branch is inserted into the Hi-C assembly scaffolds (corresponding to full-length chromosomes at this stage), otherwise the path is added to the Hi-C assembly as an unplaced scaffold.

#### Saving of scaffolds FASTA file and liftover chain file

Hi-C scaffolds are saved as a FASTA file whose header is composed of a unique ID followed by a description of the scaffolds/contigs that were used and their orientations. A sequence of 2000 *N*s is added between scaffolds. A separate liftover chain file for transfer of, e.g., annotations from the scaffolds to the Hi-C assembly is created.

### In situ Hi-C data processing

Paired-end reads were mapped and Hi-C matrices generated and corrected at restriction enzyme resolution as described above. We generated two replicates of stage 15–16 *D. melanogaster*, *D. virilis*, and *D. busckii* embryos for further analysis and one replicate of 21- to 23-h-old *D. busckii* embryos that was used for genome assembly only. Hi-C matrices of replicates were merged using “*hicSumMatrices*,” matrix bins were merged using “*hicMergeMatrixBins*,” and matrices corrected afterward using “*hicCorrectMatrix*” tools from HiCExplorer v1.8.1. TAD boundaries were called using the “*hicFindTADs*” tool from HiCExplorer with settings “–*minDepth 15000* –*maxDepth 50000* –*step 2000* –*thresholdComparisons 0.01* –*correctForMultipleTesting bonferroni*.” In total, we sequenced 45.7 M, 51.8 M, and 64.5 M useful Hi-C reads from stage 15–16 *D. melanogaster*, *D. virilis*, and *D. busckii* embryos, respectively, and 26.8 M useful Hi-C reads from 21- to 23-h-old *D. busckii* embryos (Supplemental Table S2). For validation of our TAD calling, we used Hi-C data sets from Kc167 cells (Eagen et al. 2017), which we processed as described above.

First eigenvector (PC1) corresponding to active (A) and inactive (B) compartments was computed using “hicPCA -noe1 –norm” from HiCExplorer v2.2 after removal of heterochromatic chromosome ends. Corrected Hi-C matrices at restriction fragment resolution with 50 adjacent bins merged were used, resulting in matrices with a median bin size of ∼25 kb. The correct orientation of PC1, that is, positive values corresponding to the active compartment (A) and negative values corresponding to the inactive compartment (B), was verified for each chromosome using female H4K16ac ChIP-seq data (this study).

### *D. busckii* Hi-C scaffolding

To perform *D. busckii* genome scaffolding using Hi-C data we used our 245 de novo contigs obtained by the Illumina and PacBio hybrid approach. For the assembly using HiCAssembler we used the following parameters to restrict the iterative scaffold assembly to scaffolds of 200 kb or bigger: (1) “–*min_scaffold_length 200000*” in which scaffolds of <200 kb are added after the iterative correction; (2) “–*bin_size 10000*,” which sets the Hi-C bin size to 10 kb; (3) “–*misassembly_zscore_threshold -1.0*” to control the threshold deciding if a TAD-separation score is strong enough to be considered a misassembly; and (4) “–*scaffolds_to_ignore Backbone_81/13 Backbone_60/2 Backbone_59/2 Backbone_4/1 Backbone_53/13 Backbone_24/17 Backbone_88 Backbone_103/3*.” Those scaffolds were ignored because they probably contain numerous repetitive regions. We also set the number of iterations to 3 and defined a manual list of splits defined using “*plotScaffoldsInteractively*.”

Next, we run whole-genome alignments using NUCmer (NUCleotide MUMmer of mummer v4.0.0β) with default parameters between the *D. busckii* FASTA file produced by HiCAssembler and the FASTA file for *D. melanogaster.* The chromosome names in the *D. busckii* assembly were set accordingly to the corresponding name in *D. melanogaster*.

### *D. virilis* Hi-C scaffolding

The *D. virilis* genome was sequenced and assembled into scaffolds as part of the Drosophila 12 Genomes Consortium (Drosophila 12 Genomes Consortium et al. 2007) (Ensembl Assembly GCA_000005245.1). In the dvir_caf1 scaffold assembly, joined contigs were separated by a variable number of “NNN”s in between. For the Hi-C assembly we split scaffolds that were separated with 10,000 or more *N*s as we identified misassemblies associated with these scaffolds. For the Hi-C assembly we used the following parameters: “–*min_scaffold_length 100000* –*bin_size 5000* –*misassembly_zscore_threshold -1.0* –*num_iterations 2*.” As for the *D. busckii* assembly, we used a whole-genome alignment to the *D. melanogaster* genome to assign respective names to all chromosomes.

### *D. virilis* annotation liftover

We use the Hi-C scaffolding information to create a chain file to map the available annotation of *D. virilis* (dvir-all-r1.06.gtf) (ftp://ftp.flybase.net/releases/current/dvir_r1.06/) to the new Hi-C assembly using CrossMap v0.2.5 (Zhao et al. 2014).

### Synteny block detection

To identify synteny blocks (SBs) we use LASTZ (Harris 2007) with the following parameters: “–*gfextend* –*nochain* –*gapped*,” which identifies local alignment blocks. We then chained blocks that are within 10-kb distance, have the same orientation, and contain at least four LASTZ-defined blocks. Chained results that were <4 kb or completely overlapped a bigger synteny block were removed. The 10-kb merge distance was based on the longest intron length found in flies. Defining synteny block start and end sites as synteny breakpoints, we detect 3726 and 3252 breakpoints in the *D. melanogaster* versus *D. virilis* comparison, respectively, and 3340 and 2776 breakpoints in the *D. melanogaster* versus *D. busckii* comparison, respectively. To calculate the average number of synteny breakpoints per Mb, we divided each number of synteny breakpoints by the respective genome size in Mb and then calculated their mean.

### Overlaps between SB and TAD start and end sites

To shuffle the position of TADs and SBs along the genome while keeping the same region size distribution within the same chromosomes we used “*bedtools shuffle -noOverlapping -chrom -g chrom.size*” with the appropriate chromosome sizes attributed to the “*-g*” parameter depending on the species. To calculate the overlap of SB and TAD start and end sites with SB breakpoints we extended TAD boundaries as well as SB start and end sites by 500 bp in both 5′ and 3′ directions, calculated the fraction of overlap using “*bedtools intersect*,” and checked for significance using Fisher statistics “*bedtools fisher*” with the “*-g*” parameter according to the species.

### Calculation of Jaccard similarity index

The Jaccard similarity index was defined as

$$\mathrm{Jaccard}\;\mathrm{similarity}\;\mathrm{index}=\frac{\mathrm{TAD}\;\mathrm{and}\;\mathrm{SB}\;\mathrm{intersect}\;\mathrm{length}}{\mathrm{TAD}\;\mathrm{length}+\mathrm{SB}\;\mathrm{length}-\mathrm{TAD}\;\mathrm{and}\;\mathrm{SB}\;\mathrm{intersect}\;\mathrm{length}.}$$

If one SB overlapped several TADs, we fused these TADs into one if the SB overlapped the adjacent TAD by at least 20%. This was done to account for several TADs keeping their linear order and being represented in one SB. Without this fusion of TADs, the Jaccard similarity index of one SB perfectly overlapping multiple TADs would result in a low Jaccard similarity index for each TAD, whereas the above-mentioned method results in one fused TAD with a high Jaccard similarity index.

### Aligning TADs in between species using BLASTn

Sequences of *D. busckii* and *D. virilis* TADs were aligned to the sequences of *D. melanogaster* TADs using BLASTn (blast+ v2.6.0) (Altschul et al. 1990; Camacho et al. 2009). First, a database of *D. melanogaster* TAD sequences was made using “*makeblastdb -dbtype nucl.*” Then, the sequences of *D. virilis* and *D. busckii* TADs were compared to this database and the best hit was retrieved using “*blastn -outfmt 6 -evalue 1000 -max_target_seqs 1.*” Using this method, a BLASTn bitscore was assigned to each *D. virilis* or *D. busckii* TAD compared with *D. melanogaster* TADs, reflecting how well a given TAD in one species is conserved in the other species.

### Definition of conserved TADs

*D. melanogaster* TADs giving above-median Jaccard similarity indices or bitscores from the comparison with *D. busckii* or *D. virilis* were overlapped using “*bedtools intersect -f 0.8 -r.*” The intersect of both comparisons was overlapped and resulted in the definition of 175 conserved TADs covering 11 Mb of the *D. melanogaster* genome (see Fig. 4A).

### Characterization of conserved TADs

Conserved TADs were compared with unconserved TADs and random regions. Unconserved TADs were selected based on sizes between the 0.05th and 0.95th quantiles of conserved TADs and low Jaccard and bitscores used in the intersect for the definition of conserved TADs. We selected TADs with a similar length distribution because conserved TADs are larger than average TADs (see Supplemental Fig. S6A). Random regions were defined as genomic regions with the same length distribution inside the same chromosomes as conserved TADs and are not corresponding to TADs but random genomic regions by using “*bedtools shuffle -noOverlapping -chrom.*”

Conserved TADs in *D. melanogaster* were overlapped with all genes (Ensembl Genes 92, fruit fly genes [BDGP6]) and the number of overlapping genes per kilobase was calculated. The distribution of the gene length of overlapping genes is plotted in Supplemental Figure S6B as a control.

Enrichment of NSL3 at the boundaries of conserved TADs was shown using NSL3 ChIP-seq data from *D. melanogaster* S2 cells (Lam et al. 2012). FASTQ files were mapped using Bowtie 2 (Langmead and Salzberg 2012) with default parameters. Log2fold ratio over the input sample was calculated using bamCompare (deepTools v3.0.2) with settings “*-bs 5 -ignore chrX*.”

Overlap of conserved TADs with chromatin states was calculated using chromatin states reported in *D. melanogaster* Kc cells with the accession number GSE22069 (Filion et al. 2010). The bed file was lifted over from Dm3 to Dm6 using CrossMap (Zhao et al. 2014). The significance of the difference to unconserved TADs and random regions was tested using Wilcoxon rank-sum tests (Supplemental Fig. S6C).

Enrichment of H3K4me3, H3K36me3, H3K27me3, and Hp1α in conserved TADs was shown using ChIP-seq data of 14- to 16-h-old *D. melanogaster* embryos from the modENCODE Project (modEncode accession 5096, 4950, 3955, and 3956) (Celniker et al. 2009). Data processing included sequencing quality and adaptor trimming of single-end reads using trim_galore_v0.4.5 (bioinformatics.babraham.ac.uk) with setting “*-q 5,*” mapping using Bowtie 2 v2.3.4.1 (Langmead and Salzberg 2012). The coverage of mapped reads was calculated using deepTools v3.0.2 (Ramírez et al. 2016) with settings “*-bs 25*.” Log2fold ratio over the input sample was calculated using bamCompare (deepTools v3.0.2) with settings “*-bs 25*.” Plots representing the mean and the 95% confidence interval (CI) of the log2ratio over input have been performed using deepTools “computeMatrix” followed by deepStats “dsCompareCurves” (Richard 2019).

### H4K16ac ChIP-seq experiments

ChIP of separated male and female third-instar larvae was performed as described in Valsecchi et al. (2018) using 1 µL of Anti-acetyl-Histone H4 (Lys16) Antibody (Merck Milipore, 07-329) or 0.5 µL of Histone H3 antibody mAb MABI 0301 (Active Motif, 39763).

### H4K16ac ChIP-seq data processing

We analyzed our generated H4K16ac ChIP-seq data from *D. virilis* and *D. busckii* (see paragraph before) as well as our already published H4K16ac ChIP-seq data from *D. melanogaster* (GSE109901). Data processing included sequencing quality and adaptor trimming of paired-end reads using trim_galore v0.4 (bioinformatics.babraham.ac.uk), mapping individual replicates using bwa v0.7.12 (arXiv:1303.3997v2) followed by sorting and indexing of bam files using SAMtools-1.2 (Li et al. 2009). The coverage of mapped reads was calculated using deepTools v2.0.1 (Ramírez et al. 2016) with settings “*-bs 10 –minMappingQuality 2 –normalizeTo1x {EFFECTIVE_GENOME_SIZE}.*” We defined the effective genome size as the total genome size minus the number of *N*s, which is 117 Mb, 188 Mb, and 120 Mb for *D. busckii*, *D. virilis*, and *D. melanogaster*, respectively. Log2fold ratios of merged replicates over the input sample was calculated using bamCompare (deepTools v2.0.1) with settings “*-bs 10 –scaleFactorsMethod SES.*”

### Repeat modeling and repeat masking of genomes

We first performed de novo repeat discovery using RepeatModeler (v1.0.10) (repeatmasker.org) with default settings. Afterward, we combined the de novo discovered repeats with 2385 *Drosophila* and ancestral repeats from Repbase (Bao et al. 2015). Then we run RepeatMasker v4.0.5 (repeatmasker.org) with this combined library of repeats and default settings. All three genomes were treated the same.

### Analysis of TAD boundary motif enrichment

We used a list of boundary motifs (Ramírez et al. 2018) that we have described in *D. melanogaster* to analyze and compare their enrichments at TAD boundaries in all three species using AME (McLeay and Bailey 2010) from the MEME suite v.5.0.2. Our list contains the following motifs: Beaf-32, CTCF, GAATAGAAA, GAF, Ibf, Ohler-1, Ohler-5, Ohler-6, Ohler-8, Pita, Su(Hw), Su(Hw)_short, ZIPIC, Zw5, and Ohler-8_dreme. We extracted 500 bp around all TAD boundaries in repeat-masked genomes (see paragraph before), removed sequences with more than 100 *N*s and build a second-order model of TAD boundary sequences as the background model. We used shuffled input sequences a control, average odds score as the sequence scoring method and one-tailed Wilcoxon rank-sum test as the motif enrichment test.

To compare motifs that we have described to be enriched at promoter (Ohler-1, Beaf-32, Ohler-6, ZIPIC, and Ohler-8) or nonpromoter (CTCF, Su(Hw), Ibf) boundaries (Ramírez et al. 2018) in conserved TAD boundaries versus unconserved TAD boundaries, we analyzed enrichment of these motifs using AME.

### Analysis of Beaf-32 ChIP-seq

FASTQ files for Beaf-32 ChIP-seq and input from GSM762845 (Van Bortle et al. 2014) were downloaded and aligned to the Dm3 assembly using Bowtie 2 (Langmead and Salzberg 2012). MACS2 (Zhang et al. 2008) was used to identify peaks. bamCompare and bamCoverage from deepTools2 (Ramírez et al. 2016) were used to create normalized coverage bigWig files. The processed files were lifted over from Dm3 to Dm6 using CrossMap (Zhao et al. 2014).

### Polytene chromosome spreads

Polytene chromosomes from separated male and female third instar larvae in all three *Drosophila* species were prepared as previously described (Zink and Paro 1995). Briefly, fixed and blocked polytene chromosome spreads were incubated with a homemade primary anti-MOF antibody (Mendjan et al. 2006) (1:400 in goat serum). The secondary antibody (Alexa 488 goat anti-rabbit, A-11034 from Thermo Fisher Scientific) was used in a 1:500 dilution together with Hoechst in a 1:500 dilution. Images were obtained with a Zeiss Elyra system (Carl Zeiss Microscopy) and processed using Fiji (Schindelin et al. 2012).

### roX2 ChIRP-seq data processing

ChIRP-seq data from *D. melanogaster*, *D. virilis*, and *D. busckii* was downloaded from GEO (GSE69208) (Quinn et al. 2016). Data processing included sequencing quality and adaptor trimming of single-end reads using trim_galore v0.4 (bioinformatics.babraham.ac.uk), mapping individual replicates (odd and even) using bwa v0.7.12 (arXiv:1303.3997v2) followed by sorting and indexing of bam files using SAMtools-1.2 (Li et al. 2009). The coverage of mapped reads was calculated using deepTools v2.0.1 (Ramírez et al. 2016) with settings “*-bs 10 –normalizeTo1x {EFFECTIVE_GENOME_SIZE}.*” Log2fold ratios of merged replicates (odd and even) over the input sample was calculated using bamCompare (deepTools v2.0.1) with settings “*-bs 10* –*scaleFactorsMethod SES.*” Peak calling was done using MACS2 v2.1.1.20160309 (Zhang et al. 2008) with settings “*callpeak -f BAM –qvalue 0.01 -g {effective genome size}.*”

ChIRP samples from different species were sequenced to a different depth which affects the −log10(*q*-value) of peaks called using MACS2. Because of this and to have a comparable number of roX2 peaks for further analysis, we used the “–*normalizeTo1x*” coverage files to select 250 high-confidence roX2 peaks per species (referred to as high-affinity sites [HAS]). The criteria to select those peaks were a minimum normalized coverage of 50 (meaning 50 times the enrichment over background), the highest −log10(*q*-value), and the peak needs to be present in the two replicates. The peak summit was identified as the location of the highest coverage value.

### Aggregated Hi-C contacts

We used hicAggregateContacts from HicExplorer with corrected Hi-C matrices and settings “–*vMin 1 –vMax 2 –range 300000:1000000 –numberOfBins 30 –chromosomes X –avgType mean –transform obs/exp*” to plot aggregated Hi-C contacts of high-confidence roX2 binding sites (HAS) on the X chromosome in matrices with three bins merged (∼1.7-kb bins size). Out of the selected 250 HAS, we found 246 in *D. melanogaster*, 247 in *D. virilis*, and 213 in *D. busckii* to be located on the X chromosome. We choose the respective number of random regions on the X chromosome (“*shuffleBed*” from BEDTools2) for comparison with random aggregated Hi-C contacts.

Enriched Hi-C contacts between the respective number of TAD boundaries on the X chromosome of the lowest *z*-score were visualized using aggregate plots as described above for HAS. Distances of HAS to the closest TAD boundary were added on the opposite side of the TAD boundary to get “mirrored” HAS. Aggregated Hi-C contacts were plotted as described above. Gene expression (normalized counts) analyses was performed using library size normalized RNA-seq counts from 14- to 20-h-old embryos from modENCODE data sets obtained from (Ramírez et al. 2018) and also available on the Chorogenome web server (http://chorogenome.ie-freiburg.mpg.de/).

### HAS to HAS Hi-C contact visualization

Figure 5B shows total HAS to HAS obs/exp Hi-C contacts (red arcs) displayed using pyGenomeTracks v2.0 (https://github.com/deeptools/pyGenomeTracks). The total HAS to HAS contacts were retrieved by converting Hi-C matrices at restriction enzyme sites resolution with three adjacent bins merged using “*hicMergeMatrixBins*” into obs/exp matrices with “*hicTransform*” that were then exported in GInteractions format using “*hicExport*” from HiCExplorer v2.1.4. Then anchors comprised in GInteractions files were overlapped with the HAS defined in each species using the “*InteractionSet*” R package (Lun et al. 2016).

### Analysis of phenotypic classes of alleles

The essentiality of genes overlapping conserved TADs (Fig. 4I) was assessed in *D. melanogaster* using the FlyBase automated gene summaries. The genes were filtered for genes with phenotypic annotation in *D. melanogaster* (i.e., 21 671 FBgn IDs). The analysis consists of calculating the fraction of genes displaying one of the four groups of phenotypic classes aggregated from the 184 different terms found in the automated gene summaries: the “Lethal” class corresponds to genes with “; lethal” or “lethal -” annotation, the “Increased mortality” class corresponds to genes with “increased mortality” annotation, the “Some die” class corresponds to genes with “some die during” annotation, and the “Viable” class corresponds to genes with “viable” annotation. Genes can have multiple annotated phenotypic classes as different alleles can have different phenotypic effects that can be lethal, partially lethal, or viable; thus, the fraction displayed in Figure 4I do not sum to 1.

### Gene Ontology enrichment of biological processes

Gene Ontology Biological Processes (GOBP) enrichment was computed using DAVID (Huang et al. 2009a,b) with default parameters by assigning HAS ± 30 kb overlapping genes as target, and Random ± 30 kb overlapping genes as background. We furthermore compared the same gene lists (HAS ± 30 kb and Random ± 30 kb overlapping genes) using the goProfiles R package (https://doi.org/doi:10.18129/B9.bioc.goProfiles) at the second level of the GOBP hierarchy using the “basicProfile,” “mergeProfiles,” and “plotProfiles” functions. A Holm corrected Fisher exact test has been performed using the “fisherGOProfiles” function, as suggested by the goProfiles vignette, to compare the terms enrichment between HAS ± 30 kb and Random ± 30 kb genes.

### Statistics

All statistical tests are reported in the respective figure legends. All boxplots show interquartile ranges (IQR, 0.25th to 0.75th quartile [Q_1_–Q_3_]), whiskers represent Q_1_- 1.5*IQR (bottom), Q_3_- 1.5*IQR (top), and notches represent the $\mathrm{median}\pm (1.57^{*}\mathrm{IQR}/\sqrt{n})$.

## Data access

The raw PacBio and Hi-C data have been deposited to the NCBI Sequence Read Archive (SRA) under accession numbers SRR7029387–SRR7029398. ChIP-seq and processed Hi-C data including both genome assemblies have been deposited to the NCBI Gene Expression Omnibus (GEO; http://www.ncbi.nlm.nih.gov/geo/) under accession number GSE120752.

HiCAssembler is freely available at https://github.com/maxplanck-ie/HiCAssembler.

## Supplementary Material

Supplemental Material
