# Supplementary material for: Hi-C guided assemblies reveal conserved regulatory topologies on X and autosomes despite extensive genome shuffling
Source: Genes Dev. 2019 Nov 1;33(21-22):1591–612. doi: 10.1101/gad.328971.119 (PMC6824461; doi:10.1101/gad.328971.119)
Supplement: Supplemental Material [file supp_33_21-22_1591__index.html]

Hi-C guided assemblies reveal conserved regulatory topologies on X and autosomes despite extensive genome shuffling — Supplemental Material 

# Hi-C guided assemblies reveal conserved regulatory topologies on X and autosomes despite extensive genome shuffling

## Supplemental Material

- Supplemental\_Information.pdf
